# “Is there a link between women’s empowerment and childhood vaccination?“: A multilevel analysis using the Philippines Demographic and Health Survey Data 2017 and 2022

**DOI:** 10.1186/s12889-026-27198-3

**Published:** 2026-04-06

**Authors:** Kim L. Cochon, Paul K M Poon, Josue Antonio G. Estrada, Jean H. Kim

**Affiliations:** 1 JC School of Public Health and Primary Care, Faculty of Medicine, The Chinese University of Hong Kong, Shatin, New Territories, Hong Kong SAR China; 2 Department of Clinical Epidemiology, College of Medicine, University of the Philippines - Manila, Manila, Philippines; 3 Department of Epidemiology, Boston University School of Public Health, Boston, MA USA

**Keywords:** Childhood vaccination, Women’s empowerment, Intimate partner violence, Multilevel modelling, Philippines, Low- and middle-income country

## Abstract

**Background:**

Childhood vaccination reduces child mortality, morbidity, and improves developmental outcomes. In the Philippines, where vaccination rates are low, understanding sociocultural influences on childhood vaccination is essential. Given women’s crucial role in ensuring childhood vaccination, this study investigated the association of women’s empowerment with childhood vaccination.

**Methods:**

Cross-sectional data were obtained from 6,186 partnered women aged 15–49 years who participated in the 2017/2022 Philippine Demographic and Health Surveys. Child vaccination status was assessed based on the self-reported receipt of vaccines in the Expanded Immunization Program of the Philippines. Multilevel logistic regression was conducted to assess the influence of three domains of the Survey-based Women’s emPowERment (SWPER) Index on the outcome.

**Results:**

Women’s empowerment remained high in 2017 and 2022, while complete childhood vaccination remained low, rising marginally from 65.7% to 69.3%. At the municipality level, none of the empowerment domains were associated with childhood vaccination in either year. At the individual level, higher social independence increased complete childhood vaccination in both survey years (high social independence: *aOR* = 1.54 (95% CI: 1.12–2.13) in 2017 and *aOR* = 1.58 (95% CI: 1.13–2.20) for medium and *aOR* = 2.04 (95% CI: 1.13–2.20) for high social independence in 2022). Decision-making power showed no association with childhood vaccination in either year (*p* > 0.05), while lower tolerance to violence was positively associated with the outcome in 2017 only *aOR =* 2.10 (95% CI: 1.08–4.06) for low and *aOR =* 2.42 (95% CI:1.21–4.83) for medium tolerance to violence.

**Conclusion:**

Enabling conditions for partnered women were the most important empowerment predictor of childhood vaccination, highlighting the importance of investment in gender-equitable human capital development, such as improving women’s education.

**Supplementary Information:**

The online version contains supplementary material available at 10.1186/s12889-026-27198-3.

## Background

Childhood vaccination is a cost-effective public health intervention vital to a child’s holistic, long-term development. Vaccination not only prevents child morbidity and mortality, but also improves cognitive functioning and healthy social interactions [1–3]. In turn, improved child health leads to a healthier population and a more productive economy [4]. However, complete childhood vaccination varies greatly by country. For instance, global statistics show that in 2022, 84% of children under one year of age received a third dose of diphtheria, tetanus toxoid, and pertussis-containing (DPT) vaccine, but vaccination rates ranged from 36% in Papua New Guinea to 99% in more affluent countries [5]. Moreover, vaccination dropout rates increased after the COVID-19 pandemic, while the number of children receiving zero doses of vaccines remains substantial, especially in low- and middle-income countries [6]. Most studies on child vaccination have demonstrated associations with social determinants such as maternal education, employment status, and income [7–13].

In addition to socioeconomic indicators, several studies have provided evidence of the importance of women’s empowerment on children’s health [14–16]. As such, women’s empowerment, the process of increasing women’s access and control over strategic life choices, has been receiving increasing research interest as a determinant of maternal and child health [17]. Women’s empowerment is a multidimensional construct encompassing: (1) enabling conditions such as education and access to assets, (2) instrumental agency (decision-making ability and community participation) and (3) intrinsic agency (awareness of rights and capabilities) [18]. These domains influence health decisions through mechanisms such as improved financial access, better health information uptake, and improved healthcare access. Women with higher decision-making autonomy and intrinsic agency may exhibit stronger self-efficacy in healthcare decisions and greater confidence in challenging societal norms [14, 19–22]. Studies reported a positive association between women’s agency and childhood vaccination [14–16]. Other indicators of empowerment, such as decision-making autonomy and economic participation, have also been shown to influence childhood vaccination [15]. Meanwhile, lack of empowerment, often exemplified by the acceptance of violence against women, has been negatively associated with childhood vaccination [15].

Childhood vaccine uptake in the Philippines, a low- and middle-income country (LMIC), has remained at approximately 70–80% over the past decade. This rate falls well below the national target of 95% complete vaccination coverage for children. The low vaccination levels are also exacerbated by notably high vaccine hesitancy among the populace [23, 24]. Most studies on child vaccination in LMICs focus on populations with low levels of women’s empowerment. In contrast, the Philippines, ranked 16th in gender equality according to the 2023 Global Gender Gap Report [25], has low childhood vaccination rates. Despite this, only one study has examined the link between women’s empowerment and childhood vaccination in the Philippines, finding a positive association but using an unvalidated index that lacked domain-specific insights [26]. In light of previous evidence, there is a need to explore the dynamics of childhood vaccination and women’s empowerment for an in-depth insight into its implications and substantive recommendations. The current study evaluates independent effects of three women’s empowerment domains – intrinsic agency, instrumental agency and enabling conditions – on childhood vaccination. Additionally, regional variations in childhood vaccination uptake have been noted across the country [27, 28], thus this study also examined the effects of these women’s empowerment domains at municipality and individual levels. Lastly, we explored how the COVID-19 pandemic, with its disruptions to healthcare, increased unemployment, and gender-based violence [29], influenced the relationship between women’s empowerment and childhood vaccination by comparing pre-pandemic and pandemic data.

## Methods

### Study design, sample, and data

This study involves the analysis of secondary cross-sectional data from the Philippine Demographic and Health Surveys (DHS), conducted in 2017 and 2022. These surveys employed a multistage stratified sampling design and collected data using two questionnaires: (1) the Household Questionnaire and (2) the Woman’s Questionnaire [21]. Items in the Woman’s Questionnaire were used to define the variables in this analysis.

The final analytic samples from the 2017 and 2022 Philippine DHS were composed of women aged 15 to 49 years who are married or living with a partner and who have a child aged 1 to 2 years at the time of the surveys. The sample is restricted to the subpopulation of women with partners due to the nature of measures of women’s empowerment. These measures tackle assessment of the power dynamics, relationships within a household, and gender norms in communities. The samples from both DHS only included women with complete information on relevant variables on child vaccination, women’s empowerment, and confounders. Figure S1 presents a flowchart outlining the selection process for the final analytic sample.

The study used publicly available de-identified data requested from the DHS program. The DHS received ethical review approval from the institutional board of Inner-City Fund, now known as ICF International. Meanwhile, the Survey and Behavioural Research Ethics Committee of the Chinese University of Hong Kong reviewed this secondary data analysis (Reference No. SBRE-20-894).

The authors declare that neither study participants nor the public were involved in the design, conduct, reporting, or dissemination of this research, as the analysis was based on secondary, de-identified data that precluded such involvement.

### Measures

#### Dependent variable

Complete childhood vaccination was defined in accordance with the Expanded Program on Immunization of the Philippines [26]. Using data from the Child Immunization Section of the DHS Women’s Questionnaire, vaccination status was determined based on fourteen vaccine-specific items that capture the receipt of routine childhood vaccines for the youngest living child. These items record whether the child received: one dose of BCG; three doses of DPT; three doses of OPV/IPV; and one dose of MCV or MMR, all administered before 12 months of age [11].

Each vaccine was coded as received information from vaccination cards or maternal recall, following DHS standard coding procedures. A child was classified as completely vaccinated if all the required doses across the four vaccine groups were reported as received; otherwise, the child was classified as not completely vaccinated.

#### Independent variables

The Survey-based Women’s emPowERment (SWPER) Index [18] was used to measure women’s empowerment at the municipality and individual levels. The theoretical grounding for the SWPER Index was drawn from the framework proposed by Miedema et al., and the instrument covers the following three domains of empowerment: (1) social independence representing enabling preconditions that help women achieve their goals such as access to information; (2) attitude towards violence representing intrinsic agency or women’s incorporation of gender norms related to acceptability of violence towards women under various scenarios, and; (3) decision-making power which measures instrumental agency or the extent of a woman’s participation in household decisions [30, 31]. Each SWPER domain was derived from specific DHS items using the SWPER Global index item weights, standardized to global and means and standard deviations, and categorized into low, medium, and high empowerment levels. Higher scores in each domain translate to higher empowerment. Details on the contributing DHS items and scoring procedures are summarized in Table S1.

#### Confounders

Confounders considered in the regression models included women’s age in years (15–19, 20–29, 30–39, 40–49), number of living children (1–2, 3–5, 6–8, 9 and above), wealth index (in quintiles), married or living with a partner, place of residence (urban, rural), woman intended her last pregnancy (wanted then, wanted later, wanted no more), religion (Catholic, Protestant, Islam, other Christians, other religions, none), husband’s education (no education, primary education, secondary education, higher education), and husband’s occupation (none; service, sales, agriculture, and others; managerial, professional or technical).

### Statistical analysis

Two-level logistic regression models were used to determine the association between childhood vaccination and women’s empowerment at both the individual and municipality levels. A random intercept model for municipality was included to account for clustering and was tested using likelihood ratio test. The variance of the random intercept (i.e., municipality variances) and the intraclass correlation coefficient (ICC) were reported, and model fit was assessed through AIC and BIC values. No random intercept was included at the individual level, as each woman contributed only one observation. Fixed effects for individual level and municipality level empowerment scores were modeled. Potential confounding effects were identified using a change-in-estimate criterion (> 10% change upon removal) and selected through backward elimination. Odds ratios with 95% confidence intervals were reported, and significance was set at *p* < 0.05. Data analysis was performed using StataMP version 14 for Mac.

## Results

### Sociodemographic characteristics of the final analytic sample

Table 1 shows the sociodemographic characteristics of women included in the analysis. Across both survey years, the majority of women were aged 20–39 years and were Roman Catholic. Most of the women in both surveys attained at least secondary-level education. In 2017, 61.1% of women belonged to households in the two lowest wealth quintiles, compared with 59.5% in 2022, reflecting a modest shift toward higher wealth quintiles in the later survey.

**Table 1:** Characteristics of the final analytic samples from the Philippine DHS 2017 and 2022

Characteristic	2017 (*N* = 3,415)	2022 (*N* = 2,771)
	*n* (%)	*n* (%)
Mean age in years ± SD	29.8 ± 6.7	30.6 ± 6.7
Age group		
15–19	122 (3.6%)	86 (3.1%)
20–29	1,657 (48.5%)	1,213 (43.8%)
30–39	1,320 (38.7%)	1,155 (41.7%)
40–49	316 (9.3%)	317 (11.4%)
Educational attainment		
No education	63 (1.8%)	37 (1.3%)
Primary	710 (22.8%)	414 (14.9%)
Secondary	1,680 (49.2%)	1,370 (49.4%)
Higher	962 (28.2%)	962 (28.2%)
Wealth quintile		
First (Lowest)	1,287 (37.7%)	1,009 (36.4%)
Second	799 (23.4%)	641 (23.1%)
Middle	581 (17.0%)	456 (16.5%)
Fourth	455 (13.3%)	343 (12.4%)
Fifth (Highest)	293 (8.6%)	322 (11.6%)
Religion		
Roman Catholic	2,395 (70.1%)	1,821 (65.7%)
Islam	404 (11.8%)	284 (10.3%)
Protestant	323 (9.5%)	458 (16.5%)
Other Christian	226 (6.6%)	158 (5.7%)
Other religions	55 (1.6%)	46 (1.7%)
None	12 (0.4%)	4 (0.1%)
Number of children		
11–2	1,725 (50.5%)	1,393 (50.3%)
3–5	1,328 (38.9%)	1,098 (39.6%)
6–8	293 (8.6%)	237 (8.6%)
>=9	69 (2.0%)	43 (1.6%)
Place of residence		
Urban	1,071 (31.4%)	996 (35.9%)
Rural	2,344 (68.6%)	1,775 (64.1%)

A majority of women resided in rural areas in both survey years; however, the proportion declined substantially from 68.6% in 2017 to 64.1% in 2022. Approximately 10% of women had more than five children in both surveys.

### Vaccination rates and women’s empowerment measures

Childhood vaccination in the country showed improvement between the two surveys, increasing from 65.7% in 2017 to 69.3% in 2022 (Table 2). Coverage varied across individual vaccines. Three-dose DPT coverage increased from 73.2% in 2017 to 77.6% in 2022, while three-dose polio coverage remained relatively stable (74.9% in 2017 and 75.1% in 2022). Measles vaccine coverage was lower than that of DPT and polio, at 77.4% in 2017 and 78.5% in 2022.

**Table 2:** Vaccination status of the sampled children aged 1–2 years in Philippine DHS 2017 and 2022

Vaccination status	2017 (*N* = 3,415) *n* (%)	2022 (*N* = 2,771) *n* (%)	*p*-value
Child’s vaccination status			0.010
Unvaccinated	493 (14.4%)	364 (13.1%)	
Partially vaccinated	680 (19.9%)	487 (17.6%)	
Completely vaccinated	2,242 (65.7%)	1,920 (69.3%)	
Basic vaccines received by the children			
BCG vaccine (1 dose)	2,901 (85.0%)	2,371 (85.6%)	0.497
DPT vaccine			< 0.001
1 dose	156 (4.6%)	79 (2.9%)	
2 doses	142 (4.2%)	121 (4.4%)	
3 doses	2,499 (73.2%)	2,151 (77.6%)	
Polio vaccine			< 0.001
1 dose	69 (2.0%)	106 (3.8%)	
2 doses	186 (5.5%)	142 (5.1%)	
3 doses	2,556 (74.9%)	2,098 (75.1%)	
Measles vaccine (1 dose)	2,644 (77.4%)	2,176 (78.5%)	0.298

The highest uptake was observed for BCG, which is routinely administered at birth under the Expanded Program on Immunization, with coverage of approximately 85% in both survey years.

**Fig. 1:**
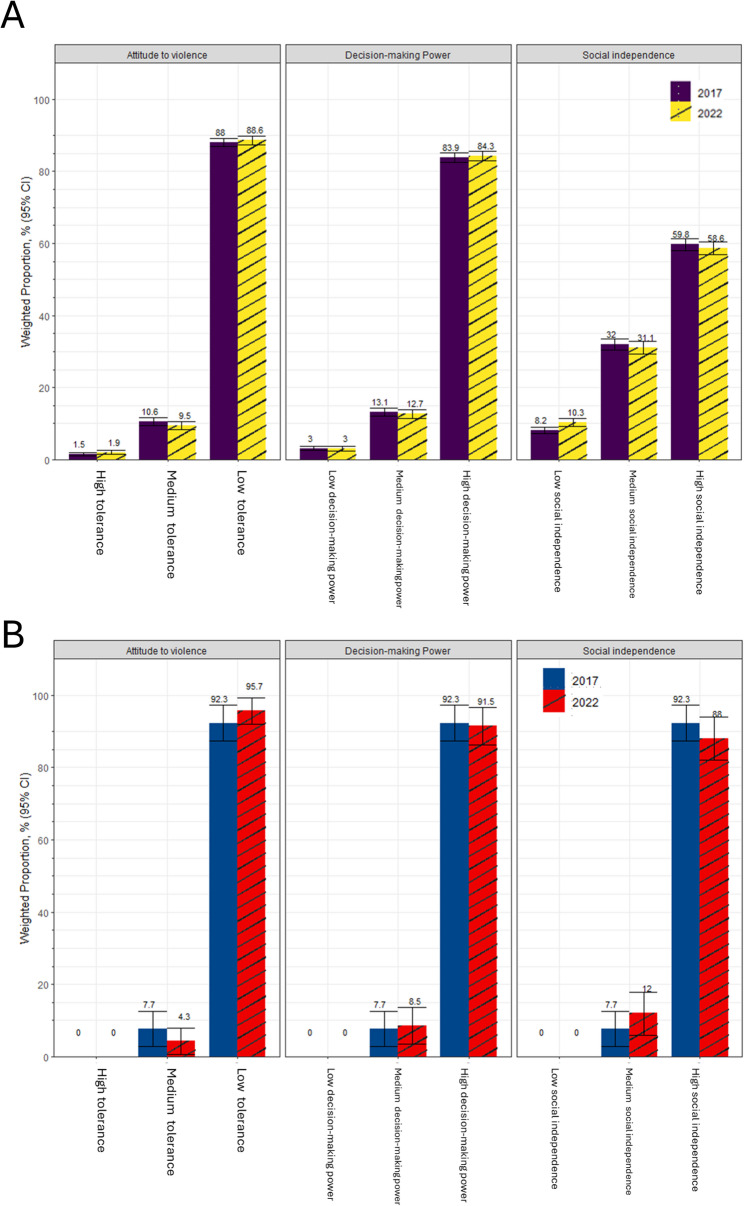
Proportion of women across levels of empowerment measured in the municipality and individual levels

A slight difference was observed in women’s empowerment in the Philippines between 2017 and 2022 (see Fig. 1). Generally, women’s empowerment in the country remained high in both years, with more than 80% of women having high decision-making power and low tolerance for violence. Meanwhile, women remained least empowered in terms of social independence in both years – with less than 60% of women having high empowerment in this domain. On the other hand, municipality level measures of women’s empowerment remained high in both years, with a marginal increase in the proportion of women with low tolerance for violence and a decrease in women with high social independence in 2022.

### Associations of SWPER domains with childhood vaccination

In both 2017 and 2022, decision-making power (instrumental agency) was not associated with childhood vaccination status at the individual level (*p* > 0.05) or municipality level (*p* > 0.05) (see Table 3). In contrast, social independence was significantly associated with complete childhood vaccination status in both years. Notably, only high social independence was associated with increased odds of complete childhood vaccination in 2017 (*aOR* = 1.54; 95% CI: 1.12–2.13), while medium social independence was not statistically significant. In contrast, both medium (*aOR* = 1.58; 95% CI: 1.13–2.20) and high social independence (*aOR* = 2.04; 95% CI: 1.44–2.89) were significantly associated with the outcome in 2022. Both low (i.e., intrinsic agency) (*aOR* = 2.10; 95% CI: 1.08–4.06) and medium tolerance to violence (*aOR* = 2.42; 95% CI: 1.21–4.83) significantly increased the odds of complete childhood vaccination in 2017, but not in 2022 (*p* > 0.05).

**Table 3:** Multilevel logistic regression analysis of the fixed effects individual and municipality level women’s empowerment on full child vaccination among children aged 1–2 years, Philippines DHS 2017 and 2022

Domains of Empowerment	DHS 2017 (*N* = 3,415)					DHS 2022 (*N* = 2,771)				
	Fully vaccinated children (%)	Unadjusted Model		Adjusted Model^a^		Fully vaccinated children (%)	Unadjusted Model		Adjusted Model^a^	
		OR (95% CI)	p-value	aOR (95% CI)	p-value		OR (95% CI)	p-value	aOR (95% CI)	p-value
Individual-level (Fixed effect)										
Attitude to violence										
Low empowerment	19 (38.0%)	Referent	-	Referent	-	25 (46.3%)	Referent	-	Referent	-
Medium empowerment	228 (63.2%)	2.72 (1.38, 5.35)	0.004	2.42 (1.21, 4.83)	0.012	168 (63.9%)	1.15 (0.56, 2.35)	0.704	0.97 (0.47, 2.02)	0.939
High empowerment	1,995 (66.4%)	2.64 (1.39, 5.02)	0.003	2.10 (1.08, 4.06)	0.028	1,728 (70.4%)	1.60 (0.82, 3.12)	0.168	1.24 (0.63, 2.46)	0.534
Decision-making power										
Low empowerment	62 (59.6%)	Referent	-	Referent	-	51 (62.2%)	Referent	-	Referent	-
Medium empowerment	322 (72.0%)	1.35 (0.80, 2.27)	0.263	1.43 (0.83, 2.45)	0.194	242 (68.8%)	0.88 (0.48, 1.60)	0.673	0.95 (0.52, 1.75)	0.867
High empowerment	1,858 (64.9%)	1.21 (0.75, 1.95)	0.429	1.21 (0.73, 1.98)	0.46	1,628 (69.7%)	1.05 (0.61, 1.81)	0.873	1.06 (0.61, 1.85)	0.840
Social independence										
Low empowerment	135 (48.2%)	Referent	-	Referent	-	147 (51.6%)	Referent	-	Referent	-
Medium empowerment	669 (61.2%)	1.51 (1.12, 2.03)	0.006	1.31 (0.96, 1.79)	0.084	556 (64.5%)	1.70 (1.24, 2.34)	0.001	1.58 (1.13, 2.20)	0.007
High empowerment	1,438 (70.4%)	2.18 (1.64, 2.90)	< 0.001	1.54 (1.12, 2.13)	0.008	1,218 (75.0%)	2.72 (2.00, 3.69)	< 0.001	2.04 (1.44, 2.89)	< 0.001
Municipality-level (Fixed effect)										
Attitude to violence										
Medium empowerment	145 (51.1%)	Referent	-	Referent	-	45 (32.9%)	Referent	-	Referent	-
High empowerment	2,097 (67.0%)	1.84 (1.00, 3.39)	0.051	1.26 (0.75, 2.12)	0.375	1,876 (71.2%)	3.25 (1.10, 9.58)	0.033	2.42 (0.90, 6.55)	0.081
Decision-making										
Medium empowerment	152 (57.8%)	Referent	-	Referent	-	165 (69.6%)	Referent	-	Referent	-
High empowerment	2,090 (66.3%)	1.31 (0.69, 2.48)	0.415	1.06 (0.61, 1.82)	0.846	1,756 (69.3%)	0.98 (0.46, 2.09)	0.959	1.19 (0.60, 2.36)	0.616
Social independence										
Medium empowerment	193 (44.1%)	Referent	-	Referent	-	213 (58.8%)	Referent	-	Referent	-
High empowerment	2,049 (68.8%)	2.66 (1.50, 4.71)	0.001	1.41 (0.86, 2.32)	0.175	1,708 (70.9%)	1.81 (0.96, 3.40)	0.067	1.38 (0.78, 2.44)	0.275
Model summary (Random effect)										
Municipality variances (SE)		0.54 (0.10)		0.54 (0.10)			0.89 (0.17)		0.84 (0.09)	
ICC		14.10%		14.10%			21.29%		20.34%	
Log likelihood^b^		-1983.20		-1964.79			-1456.24		-1421.04	
AIC		3988.40		3969.59			2934.48		2884.07	
BIC		4055.90		4092.31			2999.67		3008.54	

*aOR* means adjusted odds ratio, *CI* means confidence interval, *SE* means standard error, *ICC* means intraclass correlation coefficient

^a^ Adjusted for woman’s age, number of children, religion, wealth index, and other empowerment domains

^b^ Likelihood ratio test comparing the multilevel model to the single-level logistic regression model was statistically significant (*p* < 0.0001)

## Discussion

This analysis revealed a slight improvement in the proportion of children with complete vaccination from 2017 to 2022. However, the Philippines remains one of the countries with the lowest childhood vaccination coverage in Asia and the Pacific region [32]. Meanwhile, similar to the country’s ranking in the 2023 Global Gender Gap Report, the municipality level estimates showed that most areas have a high level of women’s empowerment in the three SWPER domains. Consequently, most women had high individual level empowerment in terms of attitude to violence (intrinsic agency) and decision-making power (instrumental agency). Enabling conditions at the individual level (social independence variable) were important in increasing childhood vaccination, whereas instrumental agency (decision-making power variable) was not associated with this outcome.

Over the past two decades, significant progress has been made in gender equality, particularly in achieving gender parity in education, economic opportunities, and other areas globally [25]. For women’s empowerment, the social independence domain is mainly explained by variables about the woman’s level of education and access to information, her age at pivotal life events (e.g., age at first birth), and the difference in age and education between her and her partner. These indicators are considered enabling conditions and resources facilitating women’s empowerment [30]. In both DHS 2017 and 2022, the mother’s level of social independence was significantly associated with the child’s vaccination, which was consistent with previous studies [16]. Ad hoc analysis of the possible moderation effect of the COVID-19 pandemic showed that individual level social independence was consistently associated with the outcome before and during the pandemic. This relationship is likely explained by the different costs of availing vaccination services, such as monetary and informational costs. Social independence may represent flexible resources such as economic opportunities, social capital, and other enabling factors that may grant mothers the ability to navigate the health system more effectively [33, 34]. For instance, the mother’s age at first childbirth, a component of the social independence variable, strongly correlates with income and wage gaps, reflecting structural and human capital differences that influence healthcare access [35].

Attitude towards violence was another empowerment domain positively associated with childhood vaccination. However, this association was observed only before the COVID-19 pandemic and not during the pandemic period. The attitude to violence variable represents intrinsic agency or the woman’s critical consciousness about her capabilities and rights and her ability to express beliefs that may not conform with prevailing norms [30]. Since the decision to vaccinate a child is usually a family affair in the Philippines that often involves the grandparents, the mother’s inherent interest in the topic of vaccination and her firm conviction about vaccinating her child strongly influence her bargaining power on this matter [36]. Compared to other household decisions, the decision to vaccinate children follows a more complicated and distinct psychosocial process that considers a lot of factors [37], as evidenced by the lack of association between decision-making power and the outcome. Furthermore, ad hoc analysis revealed that attitude to violence and decision-making power were not correlated.

Attitude to violence was not associated with childhood vaccination in 2022. This may be due to several barriers present during the COVID-19 pandemic [38]. In the absence of the pandemic, intrinsic agency, or the woman’s awareness and convictions, influenced her child’s vaccination. However, the unique circumstances brought by a public health emergency, such as the redirection of health resources towards COVID-19 patients and logistical lockdowns, may have required more than the mother’s convictions to overcome. In contrast, the role of social independence or enabling conditions in childhood vaccination was magnified in 2022 due to the importance of enabling resources, such as access to information. This demonstrates that during crises and scarcity, enabling and practical resources such as having a reliable mode of transportation may be of paramount importance and have a greater influence on healthcare utilization than intrinsic agency.

This secondary analysis has several limitations. Estimates for SWPER domain estimates —particularly for attitudes toward violence and decision-making—may be overestimated due to misreporting and social desirability bias, as interviews were often conducted in settings with limited privacy, potentially within earshot of a partner. Misclassification of children’s vaccination status is also possible, especially when based on maternal recall rather than vaccination records. In addition, the study could not account for important factors such as vaccine availability, healthcare workforce capacity, and vaccine hesitancy due to data limitations. The use of a complete-case analysis may have introduced selection bias and confounding. Nonetheless, included and excluded respondents were generally similar in age (excluded: 29.3 ± 7.4 years in 2017; 29.3 ± 6.6 years in 2022) and rural residence (2017: 67.3%; 2022: 52.7%). However, excluded women were more likely to come from lower income quintiles (Q1–Q2: 2017 = 71.2%, 2022 = 69.1%) and to have no formal education (2017: 3.9%; 2022: 1.8%). As these factors are associated with both lower empowerment and reduced vaccination coverage, their exclusion may have modestly overestimated the observed association. As a cross-sectional study, causal relationships cannot be inferred, and some level of generalizability may be limited to our target population. Findings may only be generalized to partnered women aged 15 to 49, who had children at the time of the survey. Lastly, the SWPER domains do not fully capture the multidimensional nature of women’s empowerment, including political participation, financial autonomy, and everyday constraints like time availability [39]. Despite these limitations, the study findings concerning the association between women’s empowerment and childhood vaccination were generally consistent with previous studies.

## Conclusion

This study of partnered women in the Philippines underscores the importance of women’s empowerment in the uptake of vaccination services among children. The occurrence of the COVID-19 pandemic has shown that, although women’s intrinsic agency plays a role in improving child vaccination, social independence that represents enabling conditions, such as the provision of information and improving gender equity in investments in human capital, is the most crucial in resilience to public health emergencies. Hence, efforts to improve child vaccination should be integrated into community-based and multi-sectoral programs targeting girls and women that address their needs, such as education, health literacy, sustainable livelihood, and awareness of human rights.

## Supplementary Information

Supplementary Material 1.

Supplementary Material 2.

## Data Availability

Data on the DHS can be accessed through the DHS data repository: https://www.dhsprogram.com.

## Abbreviations

aOR: Adjusted Odds Ratio
BCG: Bacille Calmette-Guérin
CI: Confidence Interval
COVID-19: Coronavirus Disease of 2019
DPT: Diphtheria, Pertussis, and Tetanus
DHS: Demographic and Health Survey
ICC: Intraclass Correlation Coefficient
IPV: Inactivated Polio Vaccine
LMICs: Low and Middle- Income Countries
MCV: Measles-containing Vaccine
MMR: Measles, Mumps, Rubella
OPV: Oral Polio Vaccine
SE: Standard Error
SWPER Index: Survey-based Women’s emPowERment Index
