# Supplementary material for: “Is there a link between women’s empowerment and childhood vaccination?“: A multilevel analysis using the Philippines Demographic and Health Survey Data 2017 and 2022
Source: BMC Public Health. 2026 Apr 6;26:1586. doi: 10.1186/s12889-026-27198-3 (PMC13191925; doi:10.1186/s12889-026-27198-3)
Supplement: Supplementary file 1 — Supplementary Material 1. [file 12889_2026_27198_MOESM1_ESM.docx]

Supplementary materials

Figure S1. Study flowchart describing how the final analytic sample was obtained.


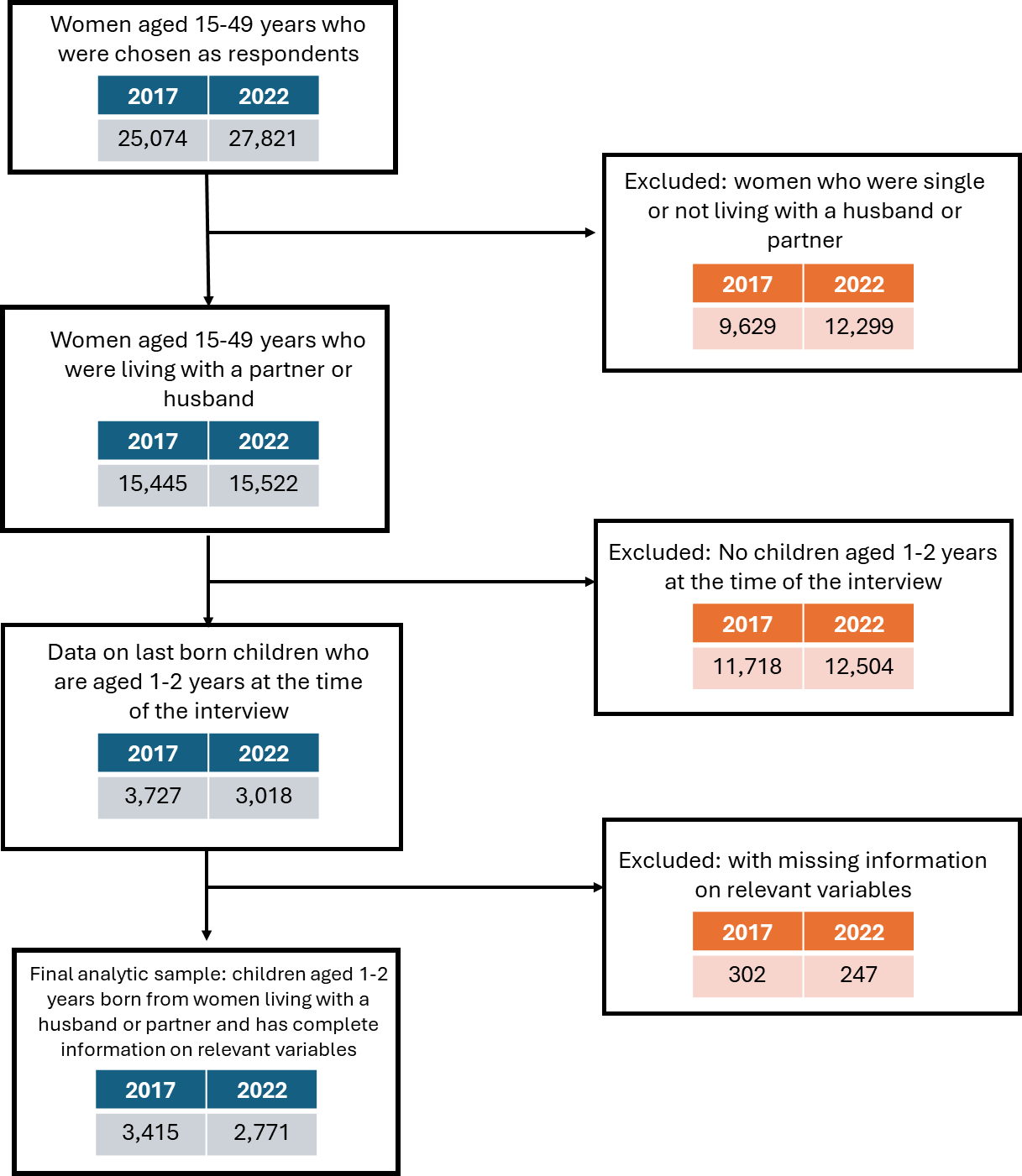


Table S1. Definition of Independent variables

| **Variable** | **Definition** | **Level of empowerment and cut-off scores** | | |
| --- | --- | --- | --- | --- |
|  |  | **Low** | **Mid** | **High** |
| Attitude to violence (Intrinsic Agency) | Pertains to the woman's opinions about wife beating and considers the statements:   - Beating is not justified if the wife goes out without telling the husband - Beating is not justified if the wife neglects the children - Beating is not justified if the wife argues with the husband - Beating is not justified if the wife refuses to have sex with the husband - Beating is not justified if the wife burns the food | ≤-0.700 | >0.700 to ≤0.400 | >0.400 |
| Social independence (Enabling conditions) | A composite variable that considers the level of education, access to information, woman's age at pivotal events, and the age and education difference between the couple. | ≤-0.559 | >-0.559 to ≤0.293 | >0.293 |
| Decision-making (Instrumental agency) | Indicates the woman's level of autonomy to decide about her healthcare, large household purchases, and visits to family or relatives. | ≤-1.000 | >-1.000 to ≤0.600 | >0.600 |

Note: The Woman’s questionnaire of the DHS was used to derive information about each women empowerment dimension. Cutoff scores were obtained by standardizing the PCA-derived scores and using the SWPER Global Index assigned global mean and standard deviations. After standardization, categorization into low, middle, and high levels was done using the SWPER Global cut-off scores since these reflect the Global SWPER scores of low- and middle-income countries.^29^
