# Supplementary material for: “Is there a link between women’s empowerment and childhood vaccination?“: A multilevel analysis using the Philippines Demographic and Health Survey Data 2017 and 2022
Source: BMC Public Health. 2026 Apr 6;26:1586. doi: 10.1186/s12889-026-27198-3 (PMC13191925; doi:10.1186/s12889-026-27198-3)
Supplement: Supplementary file 2 — Supplementary Material 2. [file 12889_2026_27198_MOESM2_ESM.pdf]

Table S1. Operational definition and computation of women's empowerment domains (SWPER Index)

SWPER domain (conceptual construct)	Main DHS items contributing to domain*	Scoring and computation	Categorization (standardized score cut-offs†)
Attitude toward violence (Intrinsic agency)	Woman's opinion on whether wife beating is justified if she:<br>● goes out without telling husband<br>● neglects children<br>● argues with husband<br>● refuses sex<br>● burns food	Individual-level scores were computed using the SWPER Global index item weights and formulae. Higher scores indicate lower tolerance to violence. Municipality-level scores were calculated as the mean of individual scores within each municipality.	Low ≤ −0.700<br>Medium > −0.700 to ≤ 0.400<br>High > 0.400
Social independence (Enabling conditions)	● Woman's education (years)<br>● Age at first cohabitation<br>● Age at first birth<br>● Frequency of reading newspapers/magazines<br>● Age difference between woman and partner<br>● Education difference between woman and partner	Individual-level scores were computed using the SWPER Global index item weights and formulae. Higher scores indicate greater social independence. Municipality-level scores were calculated as the mean of individual scores within each municipality.	Low ≤ −0.559<br>Medium > −0.559 to ≤ 0.293<br>High > 0.293
Decision-making power (Instrumental agency)	Who usually decides on:<br>● woman's healthcare<br>● large household purchases<br>● visits to family or relatives	Individual-level scores were computed using the SWPER Global index item weights and formulae. Higher scores indicate greater decision-making autonomy. Municipality-level scores were calculated as the mean of individual scores within each municipality.	Low ≤ −1.000<br>Medium > −1.000 to ≤ 0.600<br>High > 0.600

Notes: * A total of 14 DHS items were used to compute each SWPER domain. Items listed represent the main contributors; remaining items have minor loadings; † Individual-level scores were standardized using the SWPER Global index global mean and standard deviation. Cut-offs correspond to SWPER Global thresholds for low- and middle-income countries; Scores were computed following the methodology of Ewerling et al. (2020).
